# The Influence of Face Inversion and Spatial Frequency on the Self-Positive Expression Processing Advantage

**DOI:** 10.3389/fpsyg.2018.01624

**Published:** 2018-08-31

**Authors:** Yueyang Yin, Yu Yuan, Lin Zhang

**Affiliations:** ^1^School of Philosophy and Sociology, Jilin University, Changchun, China; ^2^Department of Psychology, Ningbo University, Ningbo, China; ^3^Department of Applied Psychology, School of Literature and Journalism and Communication, Changchun Guanghua University, Changchun, China

**Keywords:** expression information, face inversion, identity information, spatial frequency, the processing advantage of self-expression

## Abstract

Previous research has examined the impact of late self-evaluation, ignoring the impact of the early visual coding stage and the extraction of facial identity information and expression information on the self-positive expression processing advantage. From the perspective of the processing course, this study examined the stability of the self-positive expression processing advantage and revealed its generation mechanism. In Experiment 1, inverted self-expression and others’ expressive pictures were used to influence early structural coding. In Experiments 2a and 2b, we used expression pictures of high and low spatial frequency, thereby affecting the extraction of facial identity information or expression information in the mid-term stage. The visual search paradigm was adopted in three experiments, asking subjects to respond to the target expression. We found that under the above experimental conditions, the search speed for self-faces was always faster than that for self-angry expressions and others’ faces. These results showed that, compared with others’ expressions and self-angry expressions, self-positive expressions were more prominent and more attractive. These findings suggest that self-expression recognition combines with conceptual self-knowledge to form an abstract and constant processing pattern. Therefore, the processing of self-expression recognition was not affected by the facial orientation and spatial frequencies.

## Introduction

As an important part of the self-awareness system, self-face recognition has long attracted scholarly attention. Studies of self-faces originated from Gallup’a (1970) mirror test on chimpanzees. They found that adult chimpanzees could recognize red marks on their heads from their mirror images. Gallup believed that the accomplishment of the mirror test represents the formation of chimpanzees’ self-awareness. From an evolutionary perspective, Neisser (1997) proposed five different levels of self-awareness (ecological, interpersonal, extended, private, and conceptual self-knowledge), of which chimpanzees were in the stage of private self-knowledge. Unlike chimpanzees, human beings, with the use of tools and the development of social networks, have the ability to manipulate the symbolized self and gradually form the conceptual self. At this time, the human self-boundary has been freed from physical limitations, and it is possible to distinguish between the self and others from the perspective of the symbolized self. Self-face recognition of human beings has also combined with the conceptual self (symbolized self), meaning that self-information can be processed in an abstract and constant form (Leary and Buttermore, 2010). Compared with concrete processing, abstract processing can help individuals form accurate and rapid self-experience cognition and make competition and cooperation more effective.

Accordingly, researchers found that regardless of whether facial stimuli were upright or inverted, and whether experimental tasks were implicit or explicit, the recognition speed for self-faces was faster than for others’ faces (e.g., Tong and Nakayama, 1999; Devue and Brédart, 2011; Zahavi and Roepstorff, 2011). This result shows that the processing advantage of self-faces is stable and is not affected by facial angle and task type. In addition, previous studies have found processing advantages not only for positive expression but also for positive words, positive pictures, and other positive information, and these advantages are not affected by exposure time (Calvo and Lundqvist, 2008), low-level physical properties (Leppänen and Hietanen, 2004), or response mode (manual, voice and glance) (Calvo and Lundqvist, 2008; Calvo and Nummenmaa, 2009). These findings show that people have a processing bias toward positive information but is there a positive bias toward self-expression information? In fact, previous studies have found that processing advantages are present not only for neutral self-faces but also for emotional self-faces (Tan et al., in press). For example, Tan et al. (in press) explored the processing characteristics of self-expression, and they found that the search speed for self-expressions was faster than for others’ expression and was also faster for self-happy faces than for self-angry faces. This result is called the SPEPA. The SPEPA reflects mainly that, compared with self-negative expressions and others’ expressions, self-positive expressions are more prominent and more attractive. However, it is still unknown whether the SPEPA is stable, whether it combines with conceptual self-knowledge to form abstract processing patterns, or whether it is or is not affected by physical properties (facial angle, spatial frequency, etc.).

In a study on self-faces, the implicit association theory (IPA) proposed by Ma and Han (2010) suggests that self-face recognition is accompanied by self-concept awareness and activates positive attributes in self-concept; therefore, self-face recognition is faster than recognition of others’ faces. In accordance with the above, Sui et al. (2006) used implicit facial recognition tasks and required participants to judge the orientation of self-faces or familiar faces. They found that in the fronto-central brain area, self-faces induced greater positive waves at 220–700 ms than familiar faces. Therefore, the researchers believed that the self-face processing advantage occurred after the early structure coding, and this outcome might be caused by the differences between the evaluation of the self-face and familiar faces. Similarly, researchers used a self-concept threat priming paradigm and found that after the self-concept threat priming, the self-face processing advantage disappeared (Ma and Han, 2010; Guan et al., 2012). When self-concept was threatened, self-faces could not activate the positive attributes of self-concept, which leads to the disappearance of the self-face processing advantage. These results show that self -concept has impacts on self-face recognition. However, Tan et al. (in press) used ERP technology and discovered that the amplitudes of the N1, N2, N250, and LPP components induced by self-happy expressions were greater than those of self-angry and others’ expressions. This result shows that the SPEPA occurs in the early visual coding stage of face processing and continues to the late evaluation stage.

From the perspective of self-concept implicit activation, the self-concept threat priming paradigm indirectly examines the impact of self-concept positive evaluation on the self-information. However, this paradigm can neither examine self-expression recognition in the visual coding stage nor examine the effect of the extraction of facial identity information and expression information on the SPEPA. According to the functional model for facial recognition proposed by Bruce and Young (1986), facial recognition includes two stages. The first stage is the common structure coding stage. The second stage is the parallel processing stage of identity information and expression information. The early structural coding stage serves mainly to form facial gestalt and to provide configural information for subsequent processing. In line with the above views, researchers have found that holistic processing is very important for facial recognition, and face inversion could disrupt holistic facial processing, resulting in a face inversion effect (FIE, e.g., Yin, 1969; Farah et al., 1995; Wang and Fu, 2011). The FIE describes the greater difficulty of recognizing inverted faces than upright ones and is larger than object inversion effect (Wang and Fu, 2011). Farah et al. (1995) proposed the holistic processing theory to explain the FIE. This theory suggested that because people tend to perceive the face as a whole, the inverted face destroys the facial holistic or configural information, thereby resulting in poor performance. In contrast, object recognition is usually accomplished in a characteristic way, and thus, object inversion has less effect on recognition. In an ERP study, McCarthy et al. (1999) found that in the occipitotemporal area, the amplitudes of the N170 components induced by inverted faces were greater than those of upright faces. Many EEG studies have consistently found that the N170 (FIE-N1) components induced by inverted faces tended to have greater amplitudes and longer latencies than those of upright faces (e.g., Caharel et al., 2006; Anaki et al., 2007; Itier et al., 2007; Marzi and Viggiano, 2007; Jacques and Rossion, 2010; Boehm et al., 2011; Pesciarelli et al., 2011). The N170 component is a specific ERP component of face recognition. It is a symbol of facial structure coding and is not influenced by facial race, familiarity, gender and other factors (e.g., Bruce and Young, 1986; Rellecke et al., 2013). The effect of face inversion on the N170 component also indicated that it mainly affected the early structural coding of facial recognition. Therefore, when we used inverted facial expression materials, facial structural coding was affected.

In the second stage of face recognition, both facial identity recognition and facial expression recognition were affected by spatial frequency information (e.g., Goffaux et al., 2005; Goffaux and Rossion, 2006; Wang et al., 2011). First, White (2002) used the configural/featural change technique to examine the differences between facial identity recognition and facial expression recognition. In the experiment, the eyes moved simultaneously for configural changes, with monocular movements for featural changes, and they found that configural changes primarily affected facial identity recognition, and featural changes primarily affected facial expression recognition. Then, Goffaux et al. (2005) and Goffaux and Rossion (2006) examined the effect of spatial frequency information on facial recognition and found that LSF was related to configural processing, while HSF was related to featural processing. Based on these findings, researchers deduced that facial identity recognition was related to configural processing and depended mainly on LSF; facial expression recognition was related to featural processing and depended mainly on HSF (e.g., White, 2002; Goffaux et al., 2005; Goffaux and Rossion, 2006; Gao and Maurer, 2011; Wang et al., 2011). Although the view that facial identity recognition depends more on LSF has been relatively unanimously endorsed, researchers have questioned the view that facial expression recognition depends mainly on HSF. For example, researchers utilized fMRI technology and found that, relative to fear expressions of HSF, the amygdala responded more strongly to fear expressions of LSF. However, when subjects were asked to make subjective ratings of fear expressions, the scores of HSF were higher (Vuilleumier et al., 2003). This result suggests that facial expression recognition depends on both HSF and LSF, and they may utilize different neural channels, which have different impacts on facial expression recognition. In brain science, researchers have found that parvocellular channels transmit mainly HSF to the ventral visual cortices. The parvocellular channels has a low temporal resolution and a small visual field, but it is particularly sensitive to the objects’ length and direction. It can amplify the contrast of edges and promote the detection of edges. Therefore, the parvocellular channels perform more detailed processing of the facial expression information and are thus used primarily for slow-channel expression recognition. However, magnocellular channels transmit mainly LSF to the dorsal stream and subcortical regions (e.g., superior colliculus; pulvinar). The magnocellular channels has a high temporal resolution and a large visual field and can quickly detect facial expressions and produce a rough, holistic visual signal. Thus, it is used mainly for fast-channel facial expression recognition (e.g., Wang et al., 2011; Yang et al., 2015). According to the above analysis, we believe that facial identity recognition depends mainly on LSF, and facial expression recognition depends on both HSF and LSF. Therefore, when we used HSF facial expression materials, the extractions of identity information and fast-channel expression information were affected, while when we used LSF facial expression materials, extraction of slow-channel expression information were affected.

In summary, face inversion affected mainly the early facial structure coding, and spatial frequency information affected mainly facial identity recognition and expression recognition in the mid-term stage. From the perspective of the processing course, this study examined the impacts of face inversion and spatial frequency on the SPEPA and further revealed the SPEPA’s generation mechanism. We used inverted self-expression and others’ expressive pictures in Experiment 1 and used expressive pictures of HSF and LSF in Experiments 2a and 2b, respectively. The visual search paradigm was adopted to examine the prominence of self-expression faces in three experiments. According to previous studies, the self-face processing advantages were very stable; moreover, other self-related information (such as self-names, self-screen names, etc.) also had processing advantages (e.g., Tacikowski and Nowicka, 2010; Yang et al., 2012). Therefore, this study assumed that the SPEPA was also highly stable and was not affected by face inversion and spatial frequency information. That is, under the face inversion, HSF and LSF conditions, RTs were faster for self-expressions than for others’ expressions, and RTs were faster for self-happy faces than self-angry faces. These results suggest that not only self-face recognition but also self-expression recognition was associated with conceptual self-knowledge and formed an abstract and constant processing mode; thus, self-face recognition and self-expression recognition were not affected by facial orientation and spatial frequencies.

## Article Types

This paper belongs to the category of “Empirical Research” and should be of interest to readers in the area of “Evolutionary Psychology.”

## Experiment 1: The Influence of Face Inversion on the Spepa

In the traditional research about facial processing, the task paradigms of facial expression recognition and judgment made it difficult to highlight the expression stimulus itself, and the ecological validity of these tasks was relatively low. However, the visual search paradigm asked participants to search for target stimuli from interfering stimuli. It was possible to examine which facial stimuli were more likely to stand out among interfering stimuli and to obtain early attentional orientation (Tong and Nakayama, 1999). Therefore, we used inverted self-expressions (happy, angry) and others’ expressions (happy, angry) as our experiment materials and adopted a visual search paradigm to ask participants to recognize happy or angry expressions. By comparing the search speed of self-expressions and others’ expressions, we could observe whether the SPEPA disappeared.

### Methods

#### Subjects

Twenty-five right-handed college students (11 males and 14 females, age range: 17–20 years, *SD* = 1.16) participated in the experiment as paid volunteers. All had normal or corrected-to-normal vision. They had no history of mental illness and cerebral injury. They could correctly label happy expressions and angry expressions. Informed consent was obtained prior to the experiment. The present study was approved by the Ethics Committee of Ningbo University in accordance with the ethical principles of the Declaration of Helsinki. All subjects gave written informed consent in accordance with the ethical principles of the Declaration of Helsinki.

#### Apparatus

A Lenovo 19-inch monitor was used to present the stimuli. The screen resolution was 1024 × 768, and the refresh rate was 75 Hz. The screen background was gray, and the presented pictures were 32-bit maps.

#### Stimuli Collection and Assessment

**FIGURE 1:**
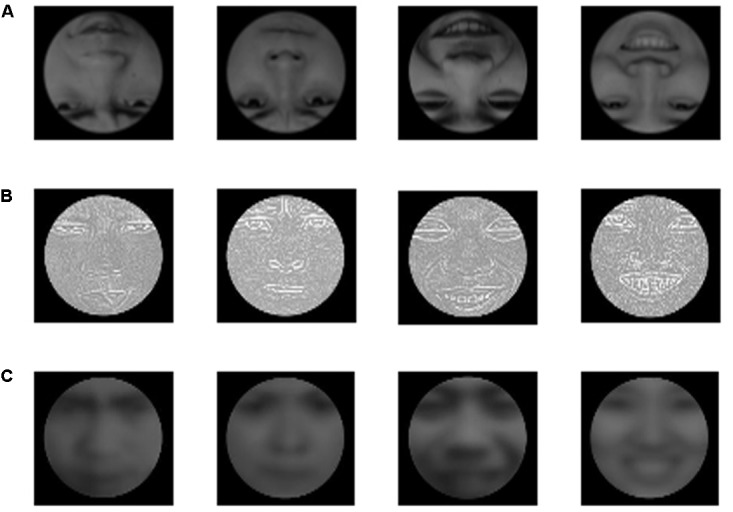
Stimulus: **(A)** inverted faces; **(B)** high-spatial frequency faces; **(C)** low-spatial frequency faces. From left to right are self-angry expression, others’ angry expression, self-happy expression, others’ happy expression.

Pictures of others’ expressions were selected from the CAFPS (Gong et al., 2011), and self-expression pictures were selected from the study by Tan et al. (in press). We obtained a total of 25 self-happy expressive pictures, 25 self-angry expressive pictures, 6 pictures of others’ happy expressions, 6 pictures of others’ angry expressions, and 12 neutrally expressive pictures (half male and half female, as filler material). Then, photos were edited in Adobe Photoshop CS6, and every picture was framed in black. The hairstyle was removed to avoid interference. Then, we rotated the self-expression and others’ expression pictures by 180° to obtain the inverted faces. Picture resolution was 472 × 545 pixels, and the bitmap was 24 bits. The brightness and contrast of the pictures were essentially the same (**Figure 1**). There were no significant differences in expressive intensity and arousal between self-expression pictures (happy and angry) and others’ expressions pictures (happy and angry, all *ps* > 0.05).

#### Experimental Design and Procedures

##### Experimental design

The experiment employed a 2 (identity type: self vs. other) × 2 (expression type: happy vs. angry) within-subject design. The dependent variables were reaction time (RT) and accuracy of expression recognition.

##### Experimental procedures

E-Prime 2.0 software was used to present the experimental stimulus and record behavioral data. In the visual search paradigm, subjects were presented with a circular search sequence (a total of six faces) and were asked to quickly and accurately determine whether there was a target expression (happy or angry) in the sequence. The distance between the screen and subjects’ eyes was approximately 60 cm. The visual angle of a single face was 2.95° × 3.04°, and the visual angle of the entire picture was 14.53° × 10.65°.

**FIGURE 2:**
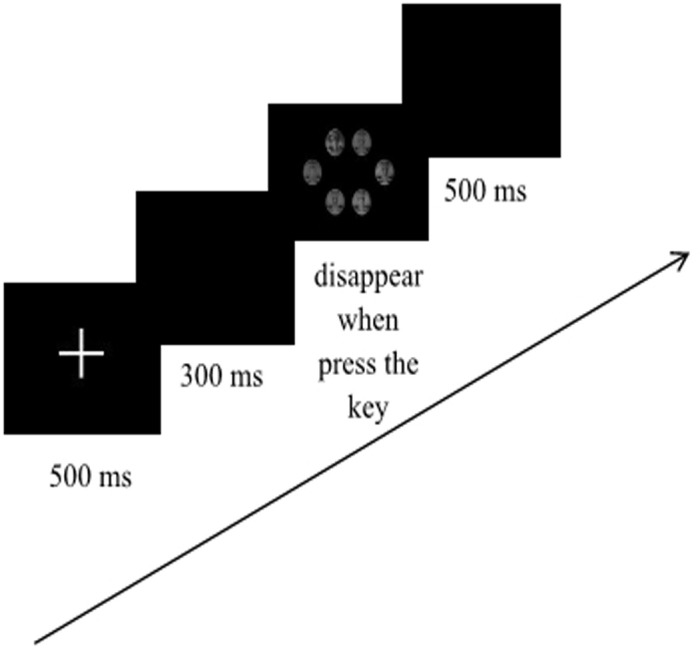
The Experiment Procedure.

In each trial of the formal experiment, all subjects performed a practice session until the accuracy of facial expression recognition reached 100% (the expression pictures used in the session were not presented in the formal experiment session). In the formal experiment, “**+**” was presented for 500 ms, followed by a blank screen for 300 ms. Then, 6 faces (1 target expressive face (happy or angry faces) and 5 neutral faces with different identities, or 6 neutral faces with different identities) were presented to subjects at the center of the screen. Subjects were asked to respond to the target expression while ignoring facial identity. They were instructed that if they observed the target expression, they were to press the “F” key on the keyboard; if the six pictures were all neutral faces, they were to press “J” key. The trial would end and the next one would automatically begin after subjects pressed a key or the picture lasted more than 3000 ms. The subjects’ response time and recognition accuracy were recorded (**Figure 2**).

The experiment comprised two blocks: happy expression recognition and angry expression recognition. The presentation sequence was counterbalanced between subjects, and the number of “F” responses was identical to the number of “J” responses. Each target face appeared in all six positions. In each position, every self-expression picture appeared 9 times, and every others’ expressions picture appeared 3 times. Previous studies have shown that the number of presentations does not affect the results (Tan et al., in press). All target expressions were presented in a random order. Each experimental condition included 54 trials; therefore, there were 432 trials in total (216 of which were filler material). A rest session was inserted every 50 trials, and the length of the rest time was determined by the subjects. The whole experiment lasted approximately 20 min for each subject.

### Result

We removed responses that exceeded ± 3 SDs and incorrect reactions, and the ratio of excluded data was 8.01%. The mean accuracies and RTs under each experimental condition are shown in **Table 1**.

**Table 1:** Response times (RTs, ms) and accuracy (%) of expression recognition (standard deviation in brackets) in Experiment 1.

Dependent variables	Self-faces		Others’ faces	
	Angry	Happy	Angry	Happy
Accuracies	96.61 (2.31)	96.33 (3.40)	87.00 (11.64)	91.28 (5.76)
RTs	986.14 (310.23)	855.68 (257.84)	1215.62 (443.37)	1008.50 (378.45)

SPSS 21 software was used to analyze data in this study. Linear mixed models (LMMs) were used to analyze data accuracy. The model specified the subjects as random variables, specifying the identity type, expression type, and the interaction between the two as fixed factors. This test revealed a significant main effect of identity type [*F*(1,24) = 28.981, *p* < 0.001] and indicated that the search accuracy was significantly higher for self-faces than for others’ faces. The main effect of expression type was not significant, *F*(1,24) = 2.156, *p* = 0.145 > 0.05. In addition, the interaction of identity and expression did not reach significance, *F*(1,24) = 2.796, *p* = 0.098 > 0.05.

Then, RTs were subjected to a 2 × 2 repeated-measures analysis of variance (ANOVA). This analysis revealed significant main effects of identity type [*F*(1,24) = 421.919, *p* < 0.001, η^2^_p_ = 0.248] and expression type [*F*(1,24) = 280.042, *p* < 0.001, η^2^_p_ = 0.179]. Specifically, the RTs were significantly shorter for self-faces than for others’ faces, and the RTs were significantly shorter for happy faces than for angry faces. In addition, there was a significant interaction of identity × expression, *F*(1,24) = 19.193, *p* < 0.001, η^2^_p_ = 0.015.

A simple effect analysis of the interaction found that in the happy expression and angry expression conditions, the RTs were significantly shorter for self-faces than for others’ faces, *F*(1,24) = 182.83, *p* < 0.001, η^2^_p_ = 0.125; *F*(1,24) = 265.22, *p* < 0.001, η^2^_p_ = 0.172. Meanwhile, in the self-face and others’ faces conditions, the RTs were significantly shorter for happy expressions than for angry expressions, *F*(1,24) = 143.04, *p* < 0.001, η^2^_p_ = 0.100; *F*(1,24) = 180.57, *p* < 0.001, η^2^_p_ = 0.124. These results showed that the SPEPA did not disappear in the face inversion condition.

## Experiment 2A: The Influence of HSF on the Spepa

Experiment 1 primarily examined the influence of face inversion on the SPEPA. The aim of Experiments 2a and 2b was to examine the influence of HSF and LSF on the SPEPA. Face inversion affected mainly the early visual coding stage, and HSF and LSF affected mainly the extraction processing of facial identity information and facial expression information. HSF affected mainly facial identity recognition and fast-channel expression information. LSF affected mainly slow-channel expression information. As in Experiment 1, Experiments 2a and 2b also adopted a visual search paradigm. By comparing the search speed of self-expressions and others’ expressions, we could examine whether the SPEPA was influenced by spatial frequency information.

### Methods

#### Subjects

The subjects of Experiment 2 were the same as in Experiment 1. To avoid the influence of experimental experience, the time interval between Experiment 2a and Experiment 1 was more than two months.

#### Apparatus

The same as in Experiment 1.

#### Stimuli Collection and Assessment

Matlab8.0 software was used for a fast Fourier transform, followed by high-pass Gaussian filtering to ensure that the cutoff frequency of all experimental stimuli was above 25c/fw and that they had equalization of treatment (Delplanque et al., 2007). The visual angle of a single face was 2.96° × 3.06°, and the visual angle of the entire picture was 19.48° × 15.66°. The remaining stimuli and assessments were the same as that in Experiment 1 (**Figure 1**).

#### Experimental Design and Procedures

The same as in Experiment 1.

### Result

We removed responses that exceeded ± 3 SDs and incorrect reactions, and the ratio of excluded data was 6.08%. The mean accuracies and RTs under each experimental condition are shown in **Table 2**.

**Table 2:** Response times (RTs, ms) and accuracies (%) of expression recognition (standard deviation in brackets) in Experiment 2a.

Dependent variables	Self-faces		Others’ faces	
	Angry	Happy	Angry	Happy
Accuracies	97.44 (2.56)	95.67 (2.98)	92.50 (4.71)	93.83 (4.34)
RTs	1225.06 (421.63)	982.24 (326.00)	1453.28.13 (479.12)	1084.33 (385.35)

As in Experiment 1, we used the LMMs to analyze data accuracy. This test revealed a significant main effect of identity type [*F*(1,24) = 20.350, *p* < 0.001] and indicated that the search accuracy was significantly higher for self-expressions than for others’ expressions. The main effect of expression type did not reach significance, *F*(1,24) = 0.088, *p* = 0.768 > 0.05. In addition, the interaction of identity and expression was significant, *F*(1,24) = 4.288, *p* = 0.041.

A simple effect analysis of the interaction found that in the happy expression condition, the search accuracy was not significantly different between self-faces and others’ faces, *F*(1,24) = 3.851, *p* = 0.061 > 0.05, η^2^_p_ = 0.138, while in the angry expression condition, the search accuracy was significantly higher for self-faces than for others’ faces, *F*(1,24) = 37.990, *p* < 0.001, η^2^_p_ = 0.613. Meanwhile, in the self-face condition, the search accuracy was significantly higher for happy expressions than for angry expressions, *F*(1,24) = 6.508, *p* = 0.018 < 0.05, η^2^_p_ = 0.213, while in the others’ faces condition, the search accuracy was not significantly different between happy expressions and angry expressions, *F*(1,24) = 1.927, *p* = 0.178 > 0.05, η^2^_p_ = 0.074.

Then, RTs were subjected to a 2 × 2 repeated-measures analysis of variance (ANOVA). This analysis revealed significant main effects of identity type [*F*(1,24) = 272.74, *p* < 0.001, η^2^_p_ **=** 0.163] and expression type [*F*(1,24) = 880.70, *p* < 0.001, η^2^_p_ = 0.386]. Specifically, the RTs were significantly shorter for self-faces than for others’ faces, and the RTs were significantly shorter for happy faces than for angry faces. In addition, there was a significant interaction of identity × expression, *F*(1,24) = 45.619, *p* < 0.001, η^2^_p_ = 0.032.

A simple effect analysis of the interaction found that in the angry expression and happy expression conditions, the RTs were significantly shorter for self-faces than others’ faces, *F*(1,24) = 222.01, *p* < 0.001, η^2^_p_ = 0.137; *F*(1,24) = 74.55, *p* < 0.001, η^2^_p_ = 0.051. Meanwhile, in the self-face and others’ faces conditions, the RTs were significantly shorter for happy expressions than for angry expressions, *F*(1,24) = 360.88, *p* < 0.001, η^2^_p_ = 0.205; *F*(1,24) = 609.14, *p* < 0.001, η^2^_p_ = 0.303. These results showed that the SPEPA did not disappear in the HSF condition.

## Experiment 2b: The Influence of LSF on the Spepa

### Methods

#### Subjects

The subjects of Experiment 2b were the same as in Experiment 1. To avoid the influence of experimental experience, the time interval between Experiment 2b and Experiment 2a was more than two months.

#### Apparatus

The same as in Experiment 1.

#### Stimuli Collection and Assessment

Matlab8.0 software was used for a fast Fourier transform, followed by high-pass Gaussian filtering to ensure that the cutoff frequency all experimental stimulus was below 6c/fw, and they had equalization of treatment (Delplanque et al., 2007). The visual angle of a single face was 2.96° × 3.06°, and the visual angle of the entire picture was 19.48° × 15.66°. The remaining stimuli and assessments were the same as that in Experiment 1 (**Figure 1**).

#### Experimental Design and Procedures

The same as in Experiment 1.

### Result

We removed responses that exceeded ± 3 SDs and incorrect reactions, and the ratio of excluded data was 5.56%. The mean accuracies and RTs under each experimental condition are shown in **Table 3**.

**Table 3:** Response times (RTs, ms) and accuracy (%) of expression recognition (standard deviation in brackets) in Experiment 2b.

Dependent variables	Self-faces		Others’ faces	
	Angry	Happy	Angry	Happy
Accuracies	96.78 (2.36)	96.50 (2.78)	92.72 (0.50)	94.61 (3.64)
RTs	1020.65 (315.39)	902.29 (287.19)	1259.79 (440.51)	1035.90 (328.29)

As in Experiment 1, we used the LMMs to analyze data accuracy. This analysis revealed a significant main effect of identity type [*F*(1,24) = 17.097, *p* < 0.01] and indicated that the search accuracy was significantly higher for self-expressions than for others’ expressions. The main effect of expression type did not reach significance, *F*(1,24) = 1.256, *p* = 0.265 > 0.05. The interaction of identity and expression was not significant, *F*(1,24) = 2.271, *p* = 0.135 > 0.05.

Then, RTs were subjected to a 2 × 2 repeated-measures analysis of variance (ANOVA). This analysis revealed significant main effects of identity type [*F*(1,24) = 500.06, *p* < 0.001, η^2^_p_ **=** 0.259] and expression type [*F*(1,24) = 379.83, *p* < 0.001, η^2^_p_ **=** 0.209]. Specifically, the RTs were significantly shorter for self-faces than for others’ faces, and the RTs were significantly shorter for happy faces than for angry faces. In addition, there was a significant interaction of identity × expression, *F*(1,24) = 45.591, *p* < 0.001, η^2^_p_ = 0.031.

A simple effect analysis of the interaction found that in the angry expression and happy expression conditions, the RTs were both significantly shorter for self-faces than for others’ faces, *F*(1,24) = 364.75, *p* < 0.001, η^2^_p_ = 0.203; *F*(1,24) = 171.16, *p* < 0.001, η^2^_p_ = 0.107. Meanwhile, in the self-face and others’ faces conditions, the RTs were both significantly shorter for happy expressions than for angry expressions, *F*(1,24) = 133.05, *p* < 0.001, η^2^_p_ = 0.085; *F*(1,24) = 293.05, *p* < 0.001, η^2^_p_ = 0.170. These results showed that the SPEPA did not disappear in the LSF condition.

To investigate whether the influence of high- and low-frequency spatial information on facial identity and facial expression was the same, we combined the data from Experiment 2a and Experiment 2b to perform 2 (self/other) × 2 (happy/angry) × 2 (HSF/LSF) repeated-measures analysis of variance (ANOVA). We found that there was significant interaction of spatial frequency × expression, *F*(1,24) = 109.92, *p* < 0.001, η^2^_p_ = 0.089. A simple effect analysis found that in the angry expression and happy expression conditions, the RTs were both significantly shorter for LSF than for HSF, *F*(1,24) = 413.81, *p* < 0.001, η^2^_p_ = 0.268; *F*(1,24) = 176.93, *p* < 0.001, η^2^_p_ = 0.135. We also found that there was marginal significance between identity type and spatial frequency, *F*(1,24) = 3.720, *p* = 0.054. Further analysis showed that in the self-expression and others’ expression conditions, both RTs were significantly shorter for LSF than for HSF, *F*(1,24) = 207.32, *p* < 0.001, η^2^_p_ = 0.155; *F*(1,24) = 418.13, *p* < 0.001, η^2^_p_ = 0.270. These results showed that both facial identity recognition and facial expression recognition were more affected by HSF.

## Discussion

From the implicit activation of self-concept, previous researchers have found that the self-face activates positive attributes in self-concept; therefore, self-face recognition is faster than others’ face recognition (e.g., Ma and Han, 2009, 2010; Guan et al., 2012). From the perspective of the processing course, this study examined the effects of early facial structure coding on the SPEPA and that of the extraction process of facial identity information and expression information of the mid-term stage, respectively. We found that whether under the condition of inverted face, or under the condition of HSF and LSF, the search speed was faster for self-faces than for others’ faces; meanwhile, the search speed was faster for self-happy expressions than for self-angry expressions. These results showed that, compared to others’ expressions and self-angry expressions, self-positive expressions were more prominent and more attractive. This finding suggests that self-expression recognition is similar to self-face recognition, which combines with conceptual self-knowledge to form an abstract and constant processing pattern. Therefore, the processing course of self-expression information is not affected by facial orientation and spatial frequencies, and thus, it has high stability.

From the perspective of evolutionary psychology, the stability of the SPEPA reflects the adaptation of human beings to their living environment. In the process of fighting against nature, human beings must pay attention to the messages from their bodies in order to adapt to their living environment. Over time, human beings have gradually developed the unconscious and automatic processing of self-related information, and this development leads to the processing of self-related information that is not limited by attentional resources. In this study, the task of facial expression recognition was used to make the subjects focus on the expression information and ignore the identity information. Under this condition, the self-identity information still had a processing advantage. Furthermore, in Experiment 1, we manipulated the face inversion to affect the facial holistic processing and then affected the extraction of identity information in the mid-term stage. In Experiment 2a, we adopted facial materials with HSF to directly influence the extraction of facial identity information. In both cases, the processing advantages of self-information still existed. This result shows that self-information is highly stable and is not limited by attentional resources. Similarly, Sui et al. (2006) used ERP technology to examine whether the self-face processing advantage was influenced by attentional resources. In the attended condition, subjects identified facial identity, while in the unattended condition, subjects identified head orientations. They found that whether in the attended or unattended condition, relative to others’ faces, self-faces induced a greater positivity over the frontocentral area at 220–700 ms. This result shows that processing advantage of self-identity information is very stable and is not limited by attentional resources.

In addition to the processing advantage of self-identity, this study also found the SPEPA. From the perspective of evolutionary psychology, the reason for the emergence of the SPEPA is that self-positive expressions can improve the attractiveness of individuals and contribute to their physical and mental health. In general, individuals who smile show high levels of optimism, openness, agreeableness and warmth, making them more attractive. Furthermore, positive expression can enhance the activity of immune cells and promote the release and synthesis of dopamine and endorphins, which are beneficial to individuals’ physical and mental health (Kashdan et al., 2014). On the other hand, from the perspective of self-concept, the self-face activated positive attributes in self-concept, and individuals were more inclined to associate positive information with themselves, thereby increasing the search speed for self-positive expression. Similarly, Epley and Whitchurch (2008) and Verosky and Todorov (2010) found that individuals were inclined to judge positive (more attractive or more trustworthy) faces as similar to their own faces. This result may be attributed to individuals’ positive expectations about their image or motivation to improve their self-esteem (Yin et al., 2015). In addition, in studies on self-emotion traits, researchers also found that individuals were more inclined to associate positive trait words with themselves and negative trait words with others (e.g., Watson et al., 2007; Fields and Kuperberg, 2015). For example, Watson et al. (2007) asked subjects to indicate whether positive and negative traits were like them or unlike them. The researchers found that RTs were significantly shorter for self-positive and non-self-negative words than non-self-positive and self-negative words. Furthermore, in studies on self-expression, Yin et al. found that after the self-concept threat was primed, the SPEPA disappeared. This result shows that self-expression recognition is also associated with self-concept. Taken together, these results show that self-information processing (including self-face recognition and self-expression recognition) is influenced by self-concept, and they are more closely related to positive self-concept. More importantly, this study found that the SPEPA remained stable after influencing early structural encoding, the extraction of expression information and identity information of the mid-term stage. This result means that individuals can process self-expression information in symbolized and abstract form, which frees it from attentional resources and facial physical properties. In other words, self-expression information have combined with conceptual self-knowledge and formed an abstract and constant processing mode. It is because of the combination with the conceptual self that the self-face has a rich meaning far beyond the face itself, and it can induce complex and diverse behaviors and emotional experiences. It is also because of the influence of the conceptual self that human beings are able to reflect on themselves, and thus, self-expression recognition shows self-positive bias. For example, the use of decoration comes from the ability of individuals to imagine how others view themselves and the willingness to improve their image and status at the symbolic level (Sedekides and Skowronski, 1997).

Self-expression recognition is associated with self-concept and includes self-evaluation. In this study, we found that the SPEPA was associated with self-positive concept and had strong stability. We speculate that this stability may be because self-expression recognition was not controlled by attentional resources. Therefore, future studies can directly explore the effect of attentional resources on the SPEPA and further explore how the self-expression of different valences is combined with conceptual self-knowledge. Furthermore, since this study only used behavioral measurements, it was impossible to measure the effects of face inversion, HSF and LSF on the processing course of self-expression recognition. Future research can examine their effects more intuitively by means of ERP technology with high temporal resolution.

## Author Contributions

YyY and LZ designed the experiments. YyY and YY made experimental materials and carried out experiments. YyY and LZ analyzed the experimental results. YyY, YY, and LZ wrote the manuscript.

## Conflict of Interest Statement

The authors declare that the research was conducted in the absence of any commercial or financial relationships that could be construed as a potential conflict of interest.

## Abbreviations

HSF: high spatial frequency
LSF: low spatial frequency
RTs: response times
SPEPA: self-positive expression processing advantage
